# Machine learning-based country-level annual air pollutants exploration using Sentinel-5P and Google Earth Engine

**DOI:** 10.1038/s41598-023-34774-9

**Published:** 2023-05-17

**Authors:** Bijay Halder, Iman Ahmadianfar, Salim Heddam, Zainab Haider Mussa, Leonardo Goliatt, Mou Leong Tan, Zulfaqar Sa’adi, Zainab Al-Khafaji, Nadhir Al-Ansari, Ali H. Jawad, Zaher Mundher Yaseen

**Affiliations:** 1 Department of Remote Sensing and GIS, Vidyasagar University, Midnapore, 721102 India; 2 New Era and Development in Civil Engineering Research Group, Scientific Research Center, Al-Ayen University, Nasiriyah, Thi-Qar 64001 Iraq; 3 Department of Civil Engineering, Behbahan Khatam Alanbia University of Technology, Behbahan, Iran; 4 Agronomy Department, Faculty of Science, University, 20 Août 1955 Skikda, Route El Hadaik, BP 26, Skikda, Algeria; 5 College of Pharmacy, University of Al-Ameed, Karbala, Iraq; 6 Computational Modeling Program, Federal University of Juiz de Fora, Juiz de Fora, MG Brazil; 7 GeoInformatic Unit, Geography Section, School of Humanities, Universiti Sains Malaysia, 11800 Penang, Malaysia; 8 School of Geographical Sciences, Nanjing Normal University, Nanjing, 210023 China; 9 Centre for Environmental Sustainability and Water Security, Research Institute for Sustainable Environment, Universiti Teknologi Malaysia (UTM), 81310 Sekudai, Johor Malaysia; 10 Department of Building and Construction Technologies Engineering, AL-Mustaqbal University College, Hillah, 51001 Iraq; 11 Civil, Environmental and Natural Resources Engineering, Lulea University of Technology, 97187 Lulea, Sweden; 12 Faculty of Applied Sciences, Universiti Teknologi MARA, 40450 Shah Alam, Selangor Malaysia; 13 Civil and Environmental Engineering Department, King Fahd University of Petroleum and Minerals, Dhahran, 31261 Saudi Arabia; 14 Interdisciplinary Research Center for Membranes and Water Security, King Fahd University of Petroleum and Minerals (KFUPM), Dhahran, Saudi Arabia

**Keywords:** Climate and Earth system modelling, Climate-change impacts, Projection and prediction, Environmental impact

## Abstract

Climatic condition is triggering human health emergencies and earth’s surface changes. Anthropogenic activities, such as built-up expansion, transportation development, industrial works, and some extreme phases, are the main reason for climate change and global warming. Air pollutants are increased gradually due to anthropogenic activities and triggering the earth’s health. Nitrogen Dioxide (NO_2_), Carbon Monoxide (CO), and Aerosol Optical Depth (AOD) are truthfully important for air quality measurement because those air pollutants are more harmful to the environment and human’s health. Earth observational Sentinel-5P is applied for monitoring the air pollutant and chemical conditions in the atmosphere from 2018 to 2021. The cloud computing-based Google Earth Engine (GEE) platform is applied for monitoring those air pollutants and chemical components in the atmosphere. The NO_2_ variation indicates high during the time because of the anthropogenic activities. Carbon Monoxide (CO) is also located high between two 1-month different maps. The 2020 and 2021 results indicate AQI change is high where 2018 and 2019 indicates low AQI throughout the year. The Kolkata have seven AQI monitoring station where high nitrogen dioxide recorded 102 (2018), 48 (2019), 26 (2020) and 98 (2021), where Delhi AQI stations recorded 99 (2018), 49 (2019), 37 (2020), and 107 (2021). Delhi, Kolkata, Mumbai, Pune, and Chennai recorded huge fluctuations of air pollutants during the study periods, where ~ 50–60% NO_2_ was recorded as high in the recent time. The AOD was noticed high in Uttar Pradesh in 2020. These results indicate that air pollutant investigation is much necessary for future planning and management otherwise; our planet earth is mostly affected by the anthropogenic and climatic conditions where maybe life does not exist.

## Introduction

### Research significant

Anthropogenic actions are the foremost reason for climatic conditions and atmospheric changes^1–3^. The new techniques like GEE platform and Sentinel-5P are used for monitoring the earth's surface change and climate change analysis applying for air quality measurement in India in addition to the global prospect^4,5^. Many types of research like air quality, AOD, air pollutant, health analysis due to air pollution, decadal change of air quality and effects on environment are conducted for analysis of the air pollutant in different methods^6–9^. The air pollution is more harmful to human life, the earth's surface, atmosphere, and overall environment^10^. Anthropogenic aerosol is the main reason for NO_2_ variation, where most of the parts are high influenced by nitrogen dioxide and CO^10–14^. The NO_2_ is related to climate change, environmental conditions, and air pollutants. Sentinel-5P is widely used for monitoring the air pollutant and Copernicus data is used for AOD monitoring^6,12,13,15,16^. In this study, air pollutants like NO_2_, CO, and chemical concentrations like AOD maps are used for monitoring the atmospheric health in India from 2018 to 2021. The air pollutant has become a serious problem gradually due to anthropogenic activities like urbanization, population pressure, industrial works, mining related activities and transportation development^17–19^. The decision making for sustainable development of human health and atmospheric activities in the earth is more important. During Covid-19 health emergencies, the air pollutants are fluctuated due to the worldwide lockdown and restriction of human activities^20^. The four different year’s air pollutants maps are helps to identifying the fluctuation of CO, NO_2_, O_3_, and SO_2_ during 2018 to 2021, where previous, during and post pandemic mean yearly air pollutants are calculated. Air pollution is more harmful for the environment as well as for the human health. Many studies are observed that the air pollutant is gradually increase over time due to the heavy transportation and industrial works^21,22^. Therefore, air pollutant studies may help to the planners and administrators for healthier disaster management and future planning. With the help of the air quality, planners can plan accordingly, where novel approaches and adaptation policies are implemented. This study generates a four years air pollutant measurement in Indian subcontinent with the help of Sentinel-5p and Google Earth Engine (GEE) platform.

### Literature review and research motivation

The clean air is furthermost significant for the human life because of oxygen and others essential gasses are generating considerable healthier life. The flora and fauna are similarly affected through several air pollutant, that’s why clean air is necessary^23,24^. The air quality maintain is the key focus of the researchers, scientists and policy-makers for sustainable planning and development of the human life in addition to the environment. One study of China indicated that the reduction of human mobility is outlandishly related to the reduction of air pollution of the 44 Chinese cities where Intercity Migration Index (IMI) was used for calculating the results^25^. The research observed that the COVID-19 lockdown also influencing factor for reducing the air pollutants in different portions of the earth surface where megacities are the indicators for human activities and air pollution related scenarios^26–31^. One study observed that the mean concentration of the NO_2_ level was reduced in the European cities where ~ 53% of the NO_2_ level are fluctuated^32^. Malaysia also affected by air pollutant and COVID-19 lockdowns were directly affected the air pollutant like PM _2.5_, NO_2_, SO_2_, and CO concentration^13^.The research studies were carryout six air pollutants in China (120 cities) from January 23, 2020 to February 29, 2020^33^. The air pollution station data can’t appropriate for identification of the distribution of country-level air quality, therefore satellite-based remote sensing data were used for monitoring the air pollutants and chemical concentration of the air^8,34^. Air pollutant measurement is more essential for environmental impact assessment; large-scale air pollutant measurement is a vital due to the large area. Therefore, GEE platform is help out for this situation (https://developers.google.com/earth-engine/datasets/tags/air-quality). Numerous algorithms were applied for calculating the air quality of an area. In India, different types of air pollutants are calculated through those notified algorithms. Earth observational Satellite established RS expertise suggestions an operative explanation on behalf of the continuing spatio-temporal observing of the air quality above the numerous measures. Machine learning approaches are widely applied for air pollutant measurement of the earth’s surface while some previous research outcomes are mentioned novel approaches for air pollutant measurement^35^.

The satellite imageries are applied for observer the air pollutant from early 1970s through the application of the Geostationary Operational Environmental Satellite (GOES), Advanced Very High Resolution Radiometer (AVHRR), and Landsat. In addition, Meteorological OPerational satellite (MetOP), Aura, and Sentinel-5 precursor (Sentinel-5P) were between the additional satellite-based datasets that have been commonly applied on behalf of air quality observing subsequently 1978. Regarding the literature, numerous investigators have deliberate observing, examining and reclamation of the air pollutants like Aerosol optical depth, SO_2_, NO_2_, CO, PM_2.5_, PM _10_, CH_4_, and O_3_ applying these RS-based satellites. During COVID-19, worldwide air pollutants are also observed to monitor the situation before, during and after COVID-19 lockdown. The GEE platform is widely used for calculating the air pollutant using Sentinel-5P and MCD19A2 data during April 2018 and 2021 in major Indian cities^36^. Sentinel-5P also used for non-linear relationship between daily and annual air pollutant like CO, NO_2_, O_3_, and SO_2_^37^. In turkey, Sentinel-5P is used for air pollutants measurement and MODIS data is used for AOD variation analysis in GEE cloud computing platform during January 2019 to September 2020^38^. The widely applied MODIS imageries were applied for calculating air pollutant in India from 2018 to 2021, where GEE platform notified formulas were applied. In India many researchers are doing the air quality measurement for environmental impact assessment and human activities analysis^10,39,40^. The air pollution is more harmful for human life because worldwide 5 millions of deaths were recorded due to air pollution and related activities. The air pollution increased temperature variation, respiratory disease, lung cancer, asthma and skin related problem^30,41^. Therefore, the research paper was used GEE cloud computing platform for delineation of the air pollutants and chemical concentration in India during 2018 to 2021. The GEE is used for hassle free large data analysis where data access, pre-processing, processing and management were done in cloud platform^42^.

### Research objectives

The air pollutant studies are located human intervention and industrials works are more harmful for development of air pollutant in India, where rural areas are located low air pollution. Megacities like Delhi, Kolkata, Mumbai, Chennai, Bangalore, Hyderabad, and Lucknow industrials areas are more air pollutant. The air pollutants were calculated form space-based Sentinel-5p data and ground measurement datasets from different pollution control board. The four different years Sentinel-5P datasets like 2018 to 2021 monthly mean data were calculated for different air pollutants estimation of India, where NO_2_, O_3_, SO_2_, and CO are measured. This study is additional important to estimating the air pollutant analysis and measurement of the air quality in India. Because numerous of the lands in India are under heavy pollution like NO_2_, SO_2_, O_3_ and CO. The chemical property like AOD is also calculated for estimating the chemical concentration of India. The anthropogenic activities, industrial works, and climatic condition phenomena are mostly affected by environmental health and public health emergencies^13,43^.The investigational outcomes are helpful for the planners, disaster management team, and administrators for protecting environment otherwise human activates and overwhelming population pressure can destroy natural environmental conditions.

##  Study area

**Figure 1:**
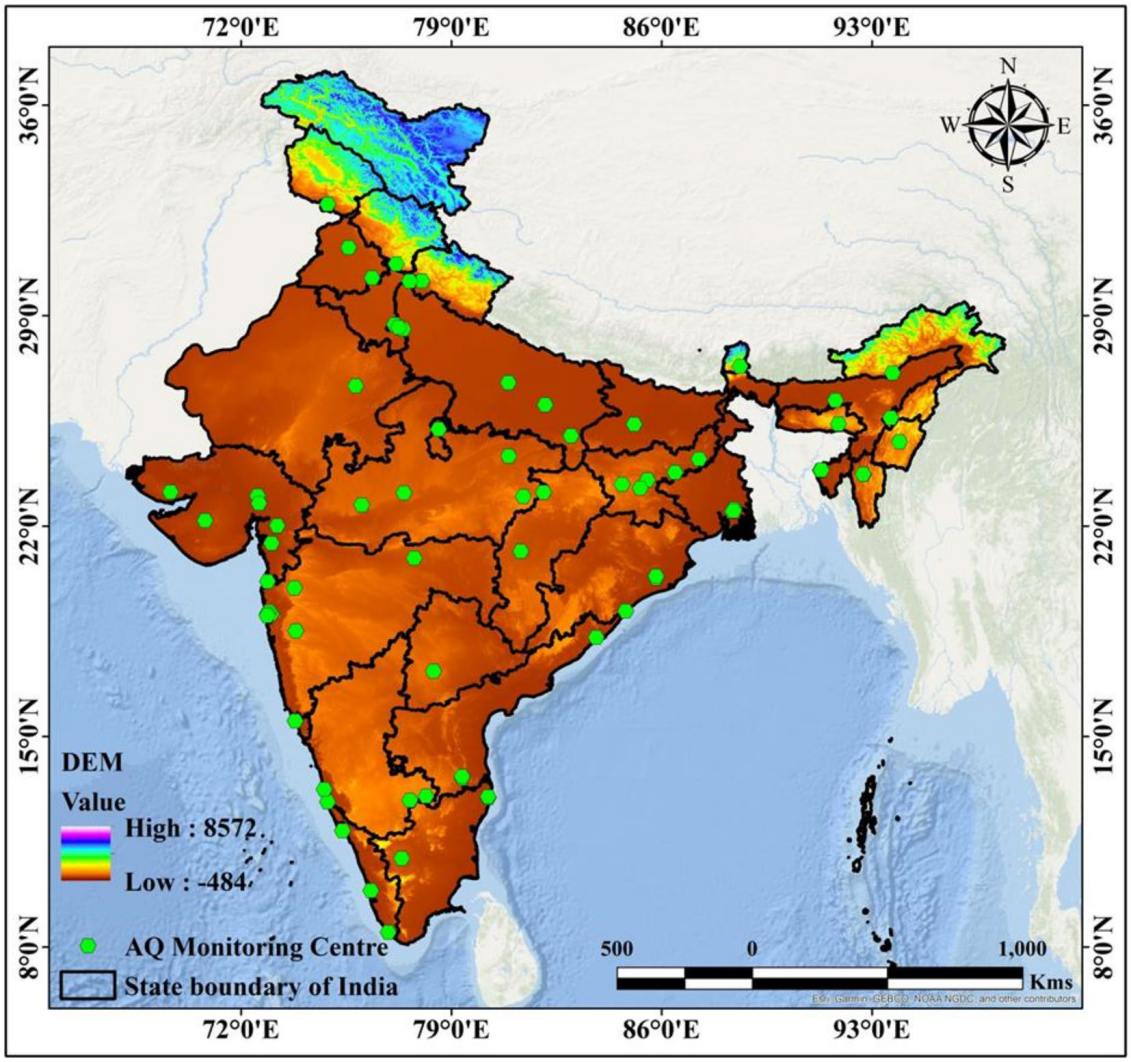
Locational Map of India and Air quality monitoring stations. India boundary map was derived from DIVA-GIS (https://www.diva-gis.org/), DEM data was derived from USGS earth explorer (https://earthexplorer.usgs.gov/) and map was prepared in ArcGIS software (https://www.arcgis.com/index.html).

The population pressure, industrial works, and anthropogenic activities like transportation development, industrial power plants, green space dynamics, and unexpected urbanization have been influencing the environment^44–46^. India is mostly affected country where, anthropogenic activities and human health-related problems are increase gradually, which is triggering factors for health issues, thermal variation, and air pollutants-related disease like asthma, respiratory disease, lung cancer, and skin-related problem. The most affected air pollutants are NO_2_, CO, O_3_, SO_2_, and CH_4_, which are affected by the air and pollutant in the environment (Fig. 1). India has mostly witnessed air pollutants-related activities in the last decades where COVID-19 times mostly influenced the air pollutants and chemical concentration of the air mass^9,29,36,47,48^. The 2020 and 2021 country-wide lockdown is the main key factor for visualization of the actual anthropogenic activities-based air pollution and related activities^36,47,48^. Because lockdown influences the air pollutant and healthy ecosystem, therefore pre, during, and post COVID-19 air pollutants measurements are the affective investigation for human activities and climate-derived scenarios.

Based on the Central Pollution Control Board, the Ministry of Environment, Forests, and Climate Change is monitoring the daily air pollutants over India (https://app.cpcbccr.com/AQI_India/) where PM _2.5_. PM _10_, NO_2_, NH_3_, SO_2_, CO, and O_3_ are measured. The station data and satellite images like MODIS and Sentinel-5P were applied for monitoring the air pollutant and chemical properties in India. The decadal maps like 2018, 2019, 2020, and 2021 datasets were much helpful for understanding the air pollution and anthropogenic activities related information, which is helpful for decision making, planning purpose, future disaster management, and research purpose. The rural areas are used biomass cake, trash, and fuel-wood for cooking purposes whereas 100 million rural households are used stoves and Turbo stoves for daily purposes. Traffic congestion, greenhouse gases emission, burning of paddy residues after harvest are more affected by air pollutants and chemical properties. Some websites are mentioned the air quality of India like India Air Quality Index (AQI) (https://www.aqi.in/dashboard/india), and World Air Quality (https://www.iqair.com/in-en/india). This research results will be helpful for future research and development where air quality investigation is more useful.

## Materials and methodology

### Data used

Air quality and chemical concentration analysis are more important for investigating the earth’s surface phenomenon and public health emergencies due to anthropogenic activities. The Sentinel-5P is widely used for monitoring the air pollutant and Copernicus data is applied for aerosol optical depth analysis. The GEE cloud computing platform is applied for air quality measurement (https://developers.google.com/earth-engine/datasets/catalog/ECMWF_CAMS_NRT) and Copernicus data is used for real time monitoring the AOD (https://apps.ecmwf.int/datasets/data/cams-nrealtime). The AOD data was derived from moderate resolution imaging spectrometer (MODIS) in the GEE platform from the year of 2018 to 2021, where pre, during and post COVID-19 air pollutants and AOD were calculated. The air quality monitoring stations data also used for validating the air pollutants and AOD values over the Indian megacities.

### Data analysis and measurement

Air quality measurement is more important for the future disaster management and planning purposes because of high industrial works, anthropogenic activities, transportation development, and some unnecessary stages^15,28,34,39^. The earth’s surface is mostly influenced by several air pollutants like NO_2_, CO, SO_2_, CH_4_, Aerosol, PM _2.5_, PM_10_, and O_3_^15,27,39,49^ The Sentinel-5P datasets are used for monitoring the air pollutant basically Nitrogen Dioxide and Carbon Monoxide^48,50^, where AOD is measured using 550 nm Copernicus near real-time monitoring data^13,48^. The code of the GEE is freely available, where the codes are modified based on the criteria and area of interest (AOI)^38,51,52^. European Space Agency (ESA) air quality monitoring data was used for air pollutant analysis (https://www.sentinel-hub.com/).

In GEE platform Sentinel-5P level 2 (L2) datasets were converted into L3 exhausting the bin_spatial procedure of harpconvert toolbox^53^. The air pollutants like NO_2_ and CO were calculated form filtered pixels with Quality Assurance (QA) standards like 75% and 50% for NO_2_ and CO respectively^54^. The source of the NO_2_ in the air is basically power plants, vehicles, industrial emissions, and anthropogenic activities^15,55^. The NO_2_ is more harmful to the people and also increased environmental health emergencies^12^. The GEE is used for monitoring the air pollutant like NO_2_ and CO, where ESA near real-time data was used for AOD in India for estimating, and investigating the environmental health during the study periods.

## Results

Air quality assessment is a very important phenomenon for investigating the anthropogenic and climatic conditions in the earth's atmosphere. The recent time like 2018–2021 is the most affected anthropogenic activities, which is more increased the air pollution like Nitrogen Dioxide, Carbon Monoxide and chemical concentration like AOD using Sentinel-5P ESA datasets, MODIS and Copernicus data. This study is to identify the air pollutant and atmospheric chemical concentration in the recent year from 2018 to 2021 pre, during and post COVID-19 periods.

### Aerosol optical depth measurement

The AOD is calculated using the GEE cloud computing platform, where the chemical composition is measured by earth observational satellite data with the near real-time monitoring system. The AOD map of India indicates the chemical properties are increased due to anthropogenic and industrial works. The block color indicates the low AOD area whereas the purple color indicates the high AOD area.

**Figure 2:**
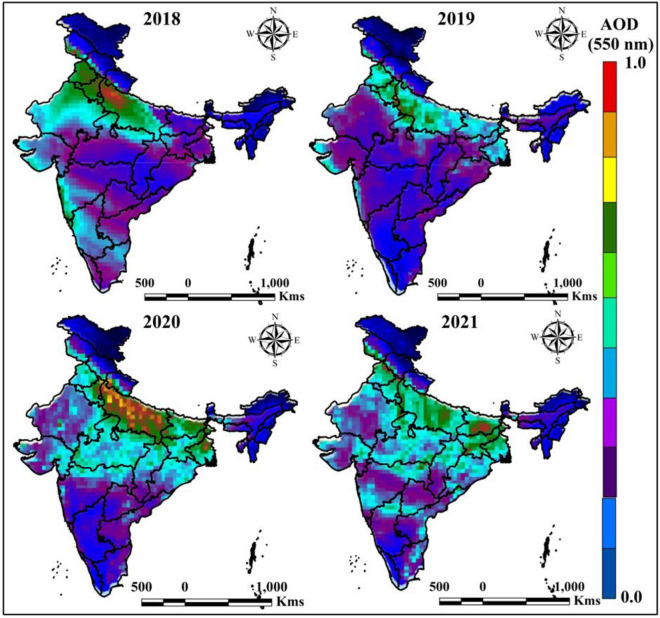
Yearly average AOD measurement of India in different time periods. India boundary map was derived from DIVA-GIS (https://www.diva-gis.org/), information was calculated in GEE platform (https://earthengine.google.com/) and map was prepared in ArcGIS software (https://www.arcgis.com/index.html).

Chemical properties of the atmosphere is more sensitive for environment in addition to the human life, therefore AOD measurement is more important. The AOD was calculated from MODIS datasets using Google Earth Engine cloud computing platform where different years mean datasets were added and calculated the values of AOD over India. Four different years’ data were used for calculating the AOD like 2018, 2019, 2020, and 2021. Figure 2 indicates the AOD maps of different time periods of India, where mostly affected AOD is located in 2018 and 2021. Uttar Pradesh Bihar, West Bengal, Jharkhand and Punjab are mostly affected by chemical concentration where rest of the area are moderate to low AOD located (Fig. 2). The AOD is fluctuated due to COVID-19 country-wide lockdown, restrictions and anthropogenic activities like industrial works, fuel burning, transportation and many others aspect. 2019 is low AOD located where 2020 and 2021 are gradually increasing the air pollutants and AOD in India. The Red color indicates high AOD values where Blue color indicates the low values. During COVID-19 pandemic air quality is improved because of low fuel use, less transportation and industrial works, therefore the AOD for different years are located variation while pre and post COVID time AOD high observed. Those phenomena indicates that the air quality variation must be fluctuated due to the industrial works in India in addition to the globally. AOD is basically measuring the aerosols from urban haze, dust particles, sea salt and some particles, which is increase in top of the atmosphere. During COVID-19 lockdown, due to the low industrial works, and less transportation urban haze, dust particles and some particles are reduce and AOD is less observed in urban and surrounding areas in India. AOD calculation indicates those phenomena from 2018 to 2021.

The earth phenomenon and environment also suffer from this decision. Only death, tears, and economic losses are the results of air pollution. The human mind is now traveling the epidemic like COVID-19, where this result is a new addition for thinking about the future. Human minds now need some surgeries to protect the human bringing. We all are birth because to help life and protect the unexpected thinks not needs any unexpected situation otherwise human life maybe lost the oxygen in future. Our motherland is now very tired, needs proper planning, management, development, and belief to protect, otherwise, the motherland earth needs hospitalization due to extreme health issues.

### Air pollutant analysis

The earth's atmosphere is mostly affected by several activities, which is frequently occurring by humans’ activities. Numerous techniques and datasets were applied for calculating and investigating the air quality in addition to the other chemical properties is generated. In this investigation, two frequently affected air pollutants like nitrogen dioxide and carbon monoxide. The air pollutant is gradually increased which shows that the human activities are truly destroying the earth’s breathing. Life is doesn’t exist without oxygen and breathing, now the earth is helpless and tried due to human activities. The study results indicate the actual condition of the earth’s atmosphere. Due to the high industrial works and vehicle use, air pollution is gradually increased in Indian megacities where dust particles, and smoke particles are more effective in human health. COVID-19 lockdown is clam down those phenomena frequently therefore pre, during and post COVID-19 air pollutant, chemical properties of air and air quality measurement is more essential for environmental sustainability.

### Nitrogen dioxide (NO_2_)

**Figure 3:**
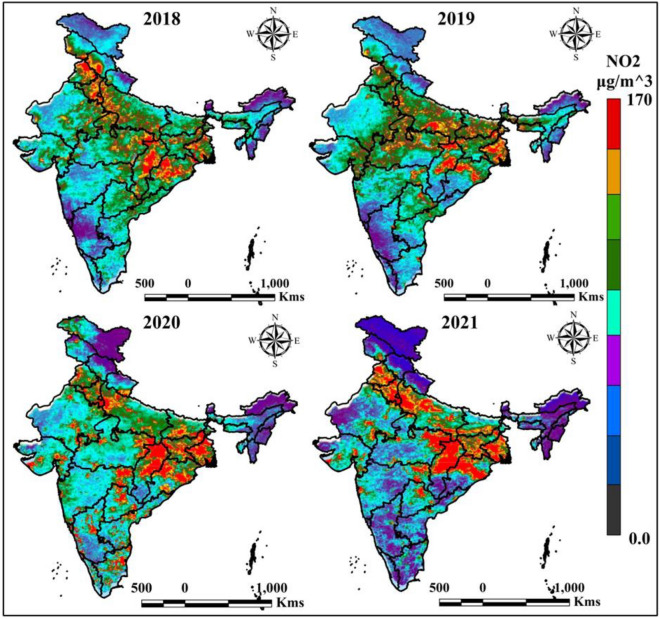
Yearly average Nitrogen Dioxide (NO_2_) measurement of India. India boundary map was derived from DIVA-GIS (https://www.diva-gis.org/), information was calculated in GEE platform (https://earthengine.google.com/) and map was prepared in ArcGIS software (https://www.arcgis.com/index.html).

The nitrogen dioxide is measured in the GEE cloud computing platform with the help of Sentinel-5P. Figure 3 indicates the NO_2_ values where red color indicates high NO_2_ values and block color indicate the low level of NO_2_. Frequently nitrogen dioxide is highly located in burning areas like the agricultural waste, fossil fuel burring location. The north Indian states like West Bengal, Jharkhand, Bihar, Odisha, Uttar Pradesh, Chhattisgarh, Madhya Pradesh, Haryana, and Punjab states are highly nitrogen dioxide located due to the agricultural waste and the fuel burring. However, the year of 2020 and 2021 were mostly nitrogen dioxide located in Delhi, Chhattisgarh, West Bengal, Haryana, and Punjab location.

Table 1 indicates the nitrogen dioxide variation of selected AQI monitoring station in different periods of India, where Kolkata, Delhi, Mumbai, Muzaffarnagar and Chennai are frequently affected through the nitrogen dioxide. The Kolkata have seven AQI motoring station where high nitrogen dioxide recorded 102 (2018), 48 (2019), 26 (2020) and 98 (2021), where Delhi AQI stations recorded 99 (2018), 49 (2019), 37 (2020), and 107 (2021). This scenarios and datasets indicate the actual condition and adaptation policies due to the anthropogenic activities and climate change condition. COVID-19 and related restrictions (lockdown, restricted transportation associability) are influences the air pollutants which indicates the climate change is depends on the anthropogenic activities and human can change the environmental issues for future life growing and sustainable healthy livelihood. Human can protect the environmental degradation and ecological diversification due to awareness, planning, management and development for sustainable life, otherwise climate change is lost the human life gradually. The NO_2_ information indicates that during COVID-19, low NO_2_ was observed, but pre phase NO_2_ is high located.

**Table 1:** Yearly mean NO_2_ of different AQI monitoring stations.

NO_2_						
Sl. no	AQI station	Parameter	2018	2019	2020	2021
1	Kolkata	Maximum	102	48	26	98
		Minimum	23	18	5	24
2	Delhi	Maximum	99	49	37	107
		Minimum	10	12	9	16
3	Patna	Maximum	57	34	29	61
		Minimum	10	9	5	12
4	Chennai	Maximum	68	57	49	62
		Minimum	24	21	12	21
5	Mumbai	Maximum	91	83	73	79
		Minimum	23	17	14	12
6	Ahmedabad	Maximum	46	39	31	50
		Minimum	12	9	8	15
7	Bengaluru	Maximum	57	49	42	62
		Minimum	11	24	21	14
8	Lucknow	Maximum	48	42	37	53
		Minimum	9	13	10	21
9	Muzaffarnagar	Maximum	105	95	82	97
		Minimum	21	14	15	13
10	Guwahati	Maximum	31	28	21	34
		Minimum	8	9	10	9

### Sulfur dioxide (SO_2_)

**Figure 4:**
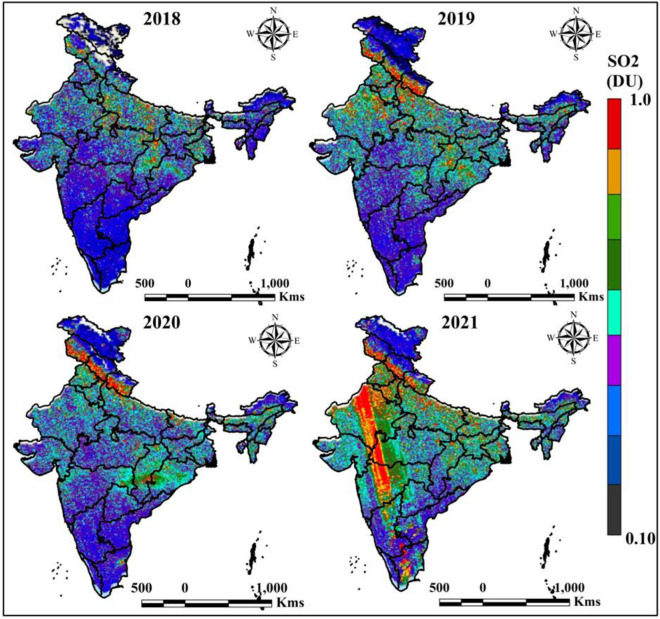
Yearly average SO_2_ variation of India in different time periods. India boundary map was derived from DIVA-GIS (https://www.diva-gis.org/), information was calculated in GEE platform (https://earthengine.google.com/) and map was prepared in ArcGIS software (https://www.arcgis.com/index.html).

The SO_2_ is most influencing air pollutants which is affected the human bodies and increased health emergencies. Figure 4 indicates the Sulfur dioxide variation of different time periods, where 2018, 2019, and 2020 is low Sulfur dioxide variation located based on 2021 data. Sentinel-5P based study of GEE platform indicates the Sulfur dioxide variation, where red color and block color indicates high and low values of Sulfur dioxide gradually. In 2019, and 2020 Uttarakhand, Jharkhand, Bihar, Jammu and Kashmir areas are mostly Sulfur dioxide located. However, the study result indicates Sulfur dioxide is highly located in 2021 map in Rajasthan, Madhya Pradesh, and Maharashtra area.

Table 2 indicates the Sulfur dioxide variation of different periods in India. 10 AQI monitoring stations are taken for general study where four mega cities of India like Delhi, Kolkata, Mumbai and Chennai are added for variation and anthropogenic activities study. The Sulfur dioxide values of Kolkata is 29 (2018), five (2019), 18 (2020) and 30 (2021), where Delhi is recorded 37 (2018), 15 (2019), 14 (2020) and 38 (2021). Those conditions indicate the restriction and proper planning can change and environmental degradation to protect our ecosystem.

**Table 2:** Yearly mean SO_2_ of different AQI monitoring stations.

SO_2_						
Sl. no	AQI station	Parameter	2018	2019	2020	2021
1	Kolkata	Maximum	29	5	18	30
		Minimum	10	3	2	4
2	Delhi	Maximum	37	15	14	38
		Minimum	5	2	4	7
3	Patna	Maximum	21	14	12	23
		Minimum	5	3	2	6
4	Chennai	Maximum	27	19	15	34
		Minimum	9	4	3	5
5	Mumbai	Maximum	34	21	18	37
		Minimum	7	6	5	8
6	Ahmedabad	Maximum	13	9	10	14
		Minimum	4	4	3	2
7	Bengaluru	Maximum	27	11	14	29
		Minimum	6	3	5	6
8	Lucknow	Maximum	28	21	19	25
		Minimum	10	5	4	7
9	Muzaffarnagar	Maximum	31	24	21	28
		Minimum	8	9	7	9
10	Guwahati	Maximum	14	11	13	17
		Minimum	2	3	2	4

### Ozone (O_3_)

**Figure 5:**
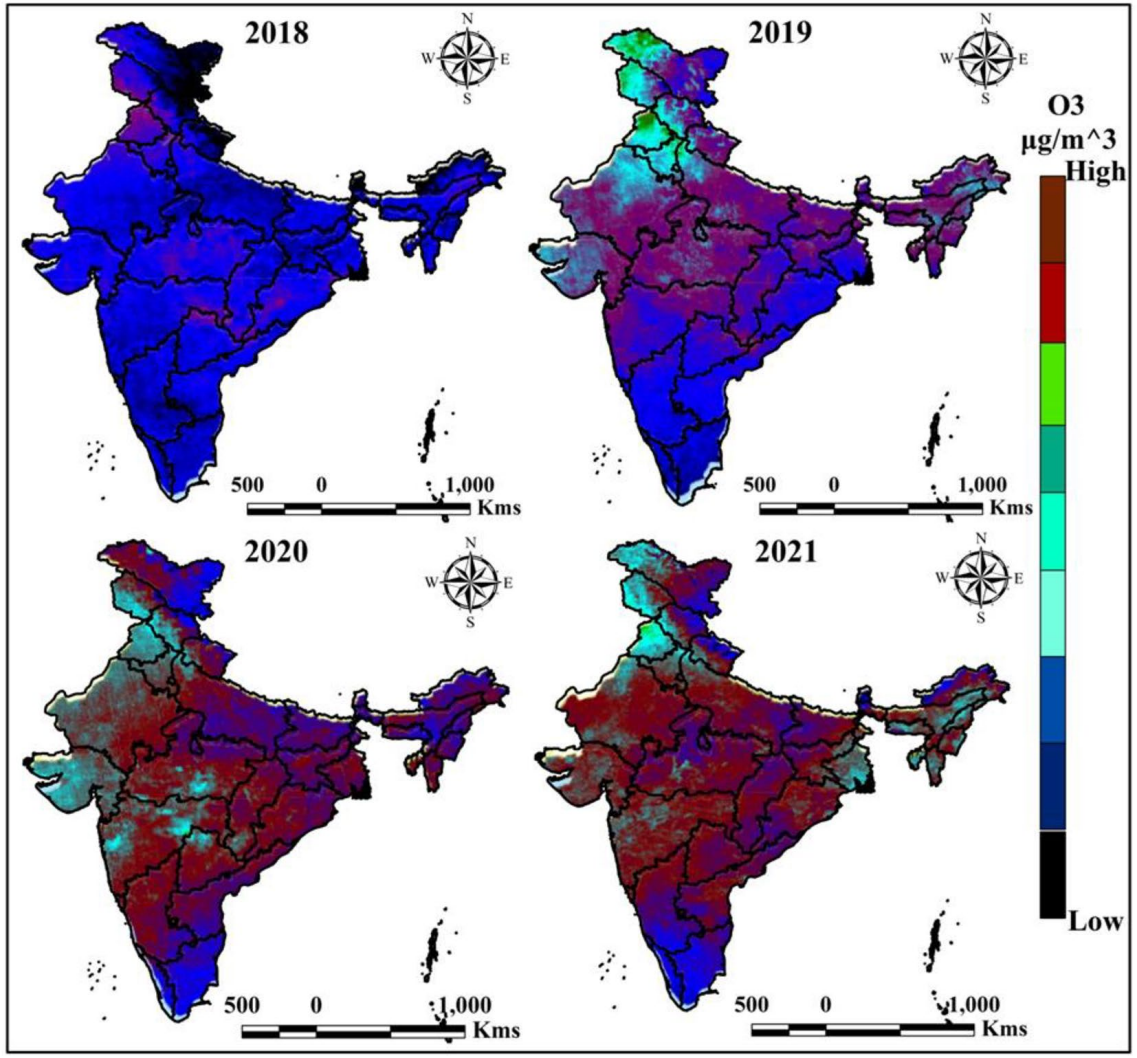
Yearly average O_3_ variation of India in different time periods. India boundary map was derived from DIVA-GIS (https://www.diva-gis.org/), information was calculated in GEE platform (https://earthengine.google.com/) and map was prepared in ArcGIS software (https://www.arcgis.com/index.html).

Ozone is more important factor for our environment where human activities and climate change are influence the ozone variation in the atmosphere. The Sentinel-5P is widely used for calculating the space-based air pollutants where ozone is more effective air pollutant which is comes from the greenhouse gasses. The ozone is mostly located in 2020 and 2021, where 2018 and 2019 are low ozone located based on Sentinel-5P and GEE platform (Fig. 5). The mostly affected states were central, north and eastern Indian states.

Table 3 indicates the variation of the ozone is different periods, where 10 AQI monitoring stations were located like Kolkata, Delhi, Patna, Chennai, Mumbai, Ahmedabad, Bengaluru, Lucknow, Muzaffarnagar and Guwahati area. This data indicates the ozone variation over the selected AQI station over India. High O_3_ damage the human health like respiratory tract tissues, irritation and body inflammation, high coughing, asthma like symptoms and chest tightness. Therefore, O_3_ measurement is more essential for environmental protection as well as healthy human life.

**Table 3:** Yearly mean O_3_ of different AQI monitoring stations.

O_3_						
Sl. No	AQI station	Parameter	2018	2019	2020	2021
1	Kolkata	Maximum	171	98	106	163
		Minimum	19	4	40	6
2	Delhi	Maximum	189	172	157	176
		Minimum	34	31	24	26
3	Patna	Maximum	107	99	85	94
		Minimum	18	12	19	14
4	Chennai	Maximum	94	84	76	104
		Minimum	24	21	15	13
5	Mumbai	Maximum	165	142	124	143
		Minimum	15	24	10	24
6	Ahmedabad	Maximum	73	62	52	79
		Minimum	14	11	9	21
7	Bengaluru	Maximum	83	79	64	89
		Minimum	28	14	11	10
8	Lucknow	Maximum	129	118	104	127
		Minimum	24	17	9	16
9	Muzaffarnagar	Maximum	143	124	117	134
		Minimum	21	9	18	12
10	Guwahati	Maximum	76	49	37	59
		Minimum	15	13	11	15

### Carbon monoxide (CO)

**Figure 6:**
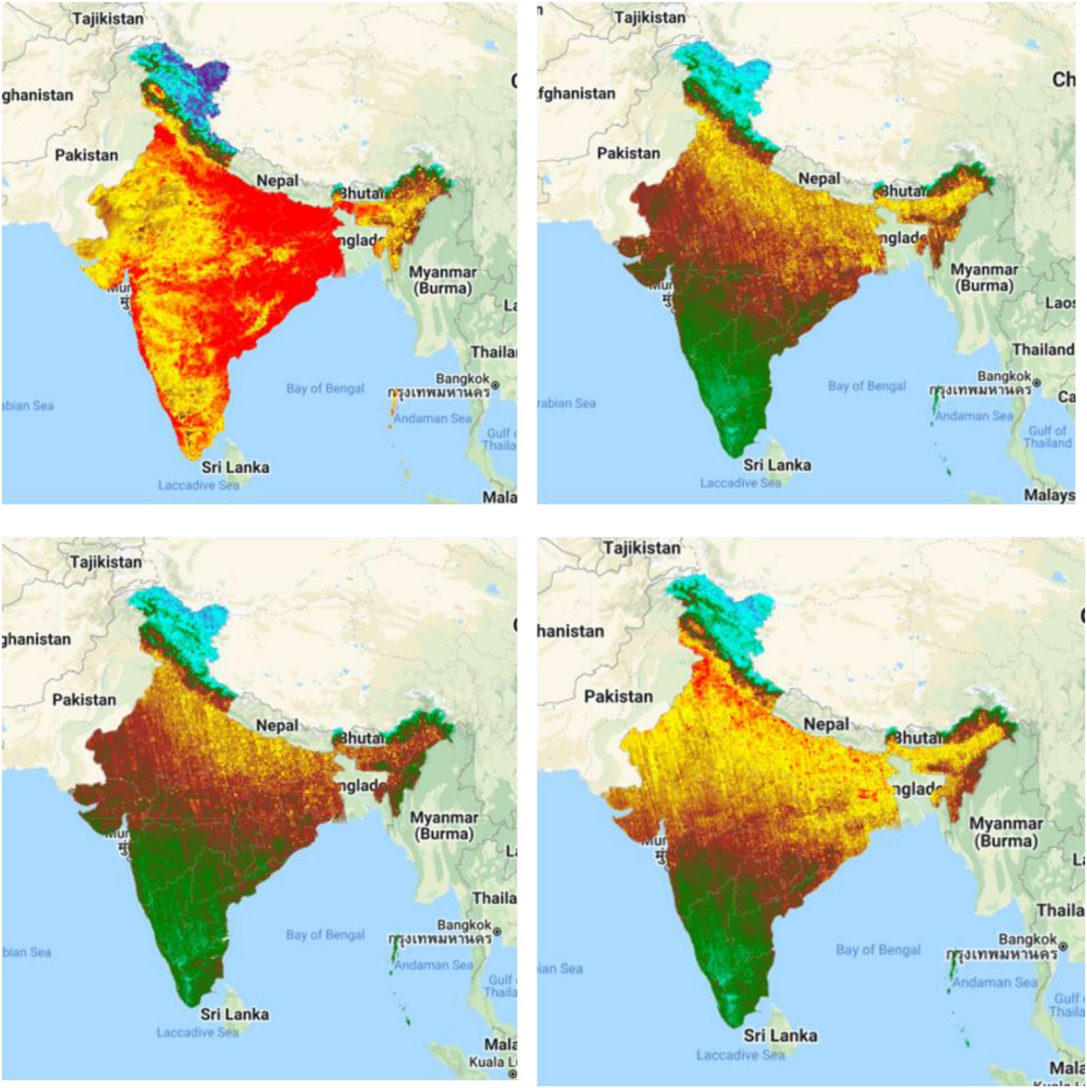
Yearly average CO variation of India in different time periods. India boundary map was derived from DIVA-GIS (https://www.diva-gis.org/), information was calculated in GEE platform (https://earthengine.google.com/) and map was prepared in ArcGIS software (https://www.arcgis.com/index.html).

The same cases are notified in the carbon monoxide maps because this map also indicates the carbon sequences are high. Figure 6 indicates the CO map of the study area where red color indicates the high CO and black color indicates low CO. This map indicates the actual earth atmospheric condition in the study area, where the whole world may face the problems like social, political, economic, and medical emergencies. CO is basically generated due to transportation activities and industrial work. The initial year like 2018 is most CO located due to anthropogenic activities and industrial work, where 2019, 2020 low CO values are were recorded due to COVID-19 restriction and lockdown. The transportation and industrial works have been stop and the results are low CO. However, in the year of 2021, CO gradually increased in the different parts of India due to reopening the industrial works, vehicles and anthropogenic activities. People can change the environmental degradation for adopting some plan and management system otherwise climate change grass the entire life. Mainly vehicles like cars, tracks, fossil fuel burn and individual vehicles are the reason for high CO variation, therefore COVID-19 time measurement of CO is more important because lockdown reducing the CO while pre COVID-19 CO is high. Needs group vehicles use, solar power applies, natural gasses apply and plantations are the possible solution to reducing CO over India.

Table 4 indicates the variation of CO of different AQI stations where mostly affected stations are Kolkata, Delhi, Mumbai, Lucknow, and Muzaffarnagar. The results indicate high values of CO recorded in Kolkata are 81 (2018), 27 (2019), 30 (2019), and 74 (2021), where Lucknow recorded 87 (2018), 72 (2019), 59 (2020), and 97 (2021). Those scenarios noticed that the air pollutant like carbon monoxide (CO) is mostly high recorded in 2018 and 2021, where during COVID-19 low carbon monoxide (CO) data were recorded.

**Table 4:** Yearly mean CO of different AQI monitoring stations.

CO						
Sl. no	AQI station	Parameter	2018	2019	2020	2021
1	Kolkata	Maximum	81	27	30	74
		Minimum	24	6	9	26
2	Delhi	Maximum	89	72	62	91
		Minimum	21	13	10	24
3	Patna	Maximum	48	39	24	52
		Minimum	9	11	9	13
4	Chennai	Maximum	53	47	31	57
		Minimum	10	9	18	15
5	Mumbai	Maximum	75	64	42	81
		Minimum	26	24	13	21
6	Ahmedabad	Maximum	37	31	29	46
		Minimum	10	16	12	14
7	Bengaluru	Maximum	52	47	39	59
		Minimum	14	12	10	19
8	Lucknow	Maximum	87	72	59	97
		Minimum	23	12	9	20
9	Muzaffarnagar	Maximum	86	74	42	83
		Minimum	15	10	16	15
10	Guwahati	Maximum	31	29	24	37
		Minimum	17	14	5	12

### Air quality index (AQI)

**Figure 7:**
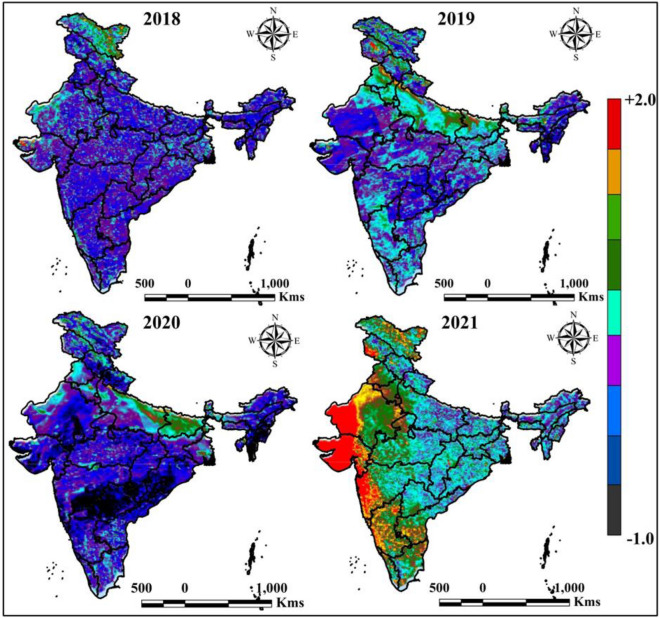
Yearly average AQI variation of India in different time periods. India boundary map was derived from DIVA-GIS (https://www.diva-gis.org/), information was calculated in GEE platform (https://earthengine.google.com/) and map was prepared in ArcGIS software (https://www.arcgis.com/index.html).

Air quality is measured by different air pollutant like PM _2.5_, PM _10_, NO_2_, SO_2_, O_3_, CH_4_, and CO in the air. The Sentinel 5P is widely used for monitoring the space-based AQI in GEE platform. Figure 7 indicates the variation of AQI in different periods, where 2018, 2019, 2020 and 2021 yearly average data were taken to calculate the variation of AQI. The 2020 and 2021 results indicate AQI change is high where 2018 and 2019 indicates low AQI throughout the year.

**Figure 8:**
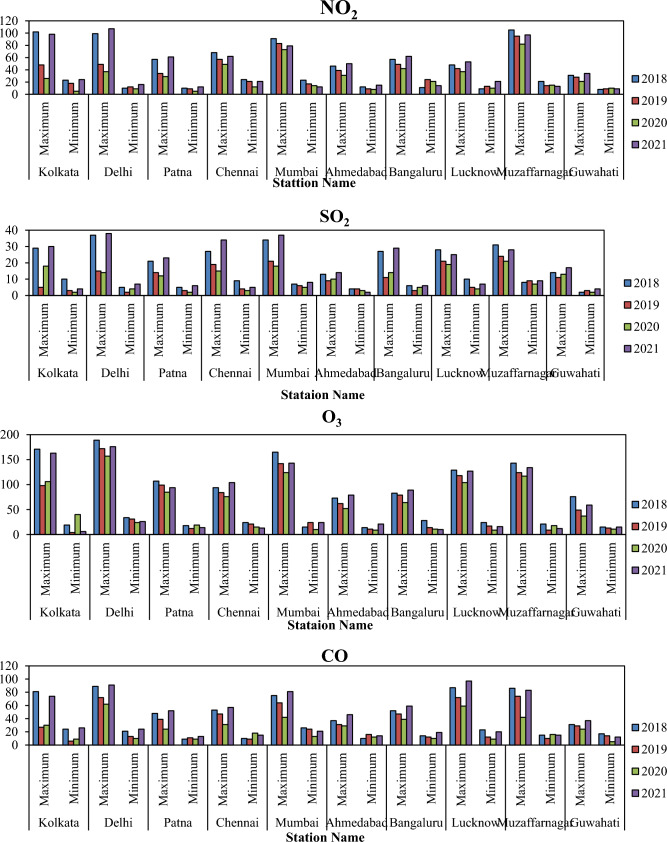
Yearly minimum and maximum air pollutants of selected AQI monitoring station in India (Source: CPCB). Information was prepared in MS Excel. Datasets were retrieved from CPCB (https://cpcb.nic.in/).

**Figure 9:**
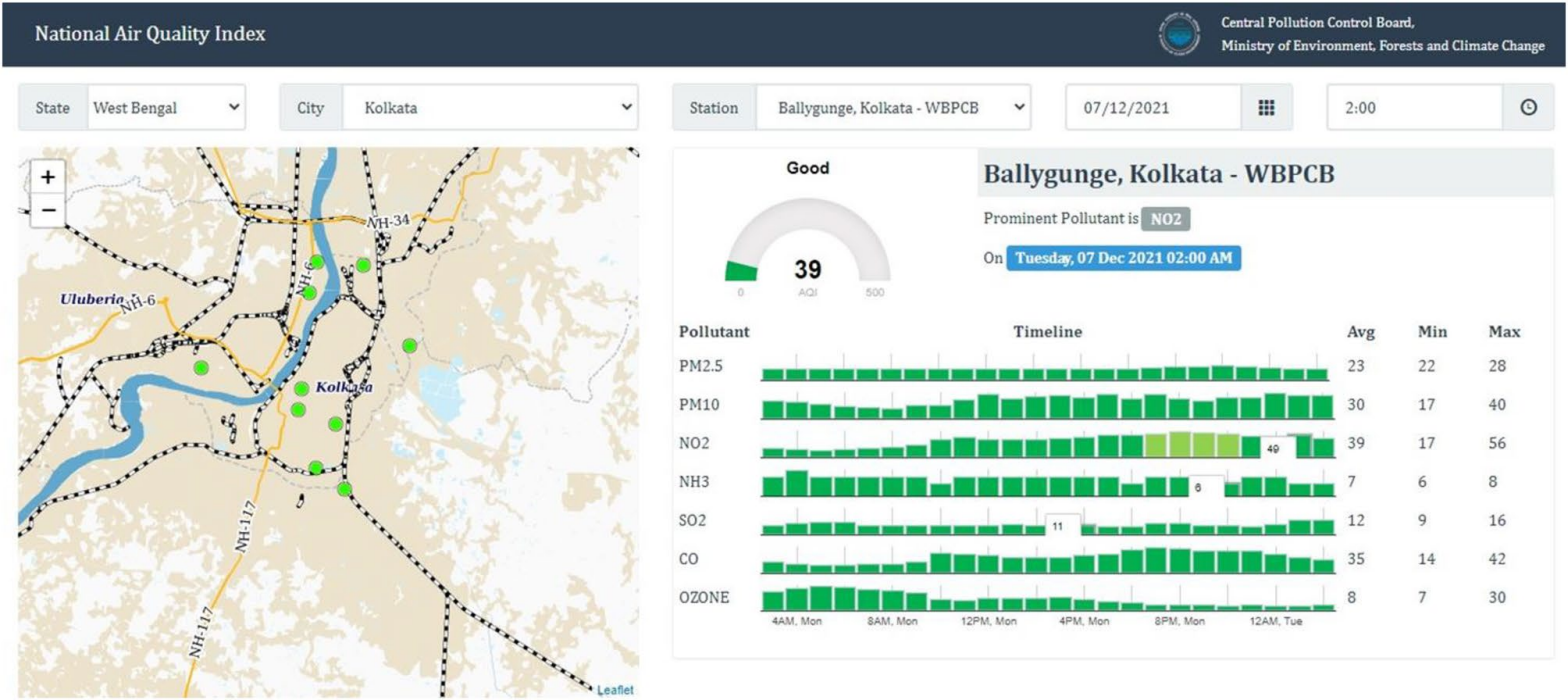
Air quality monitoring station and different air pollutants measurement units in Kolkata station, India (Source: CPCB).

Different station data were used for monitoring the variation of different air pollutants in India, where NO_2_, SO_2_, O_3_, and CO are monitoring in different ten AQI station. Figure 8 indicates the variation of maximum and minimum air pollutant of different time periods in India. This variation located that the COVID-19 restrictions and lockdowns are mostly influences the fluctuation and low air pollutants where reopening of industrial works and anthropogenic activates are increased the air pollutants in India. Figure 9 indicates that the AQI monitoring station of most effective place Ballygunge, Kolkata, where AQI and others air pollutant parameters are observed (Fig. 9).

**Figure 10:**
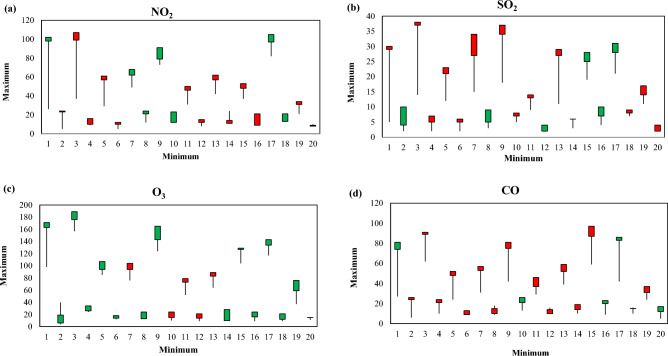
Box plot of the air pollutants in different time periods (based on tables). Information was prepared in MS Excel.

Figure 10 indicates the box plot of different AQI station minimum and maximum data where four air pollutants are recorded and investigated. The study results will helpful for planning, development, awareness and future disaster management system. People awareness is necessary for unexpected development of air pollutants otherwise climate change and anthropogenic activities are hammering the ecological diversity and destroying the natural phenomena of earth.

## Discussion

Air pollution and the extreme climatic conditions are simultaneously related to each other’s, because climate change has been triggering factor for air pollutant and chemical properties of the air. Sunlight is affected by air pollutant like methane, ozone, and aerosol, therefore those air pollutant measurements is necessary for investigating the climate change impacts on the earth’s surface. The high voltage electric discharge has been changed the oxygen to ozone where ozonosphere damaged increased the ultraviolet rays in earth’s surface. The climate change influences the air quality and pollutants where environment and ecological disturbances are noticed. India has witness of air pollutants in the different megacities where COVID-19 lockdowns were the affective and eye opening time where people can noticed the anthropogenic activities is the main reason for air pollution. Delhi, Kolkata, Mumbai, Hyderabad, Bangalore, Durgapur, Bokaro, and many others Indian cities are mostly recorded low air pollution. The different years Sentinel-5P and MODIS data were used for delineating the air pollutants and AOD in the years of 2018 to 2021. Not only India, others countries have noticeable change located during the COVID-19 country-wide lockdown and restriction^56–59^. Those conditions increase some awareness, knowledge; environment protraction related activities and planning are increase. COVID-19 triggering the low air pollution and positive air quality because of the countrywide lockdown, restrictions and transportation restrictions over India, therefore vehicles, fossil fuel use and industrial waste were not generated. Those conditions are triggering the ecological variation, environmental protection and improved air quality over India. Most of the megacities are observed low dust particles, air pollution and smoke particles. Therefore, this study can help to identifying the reason and benefits of lockdown or awareness to protect our environment. Fossil fuel also applied in the rural areas for cooking purpose but industrial areas, and vehicles are more used fossil fuel, due to the restriction, those triggering factors are reducing and increase healthy life with improved environmental condition. Nowadays many approaches were applied for air quality measurement and environmental impact assessment like machine learning^60^ where symmetric mean average percentage error (SMAPE) multicascade space–time learning model (MCST-Tree) was applied for PM _2.5_ distribution measurement. Another air pollutant like PM _2.5_, PM_10_, and O_3_ was measured through multi-AP learning network^61^. These research outcomes are mentioned the air pollution is gradually impacts on the earth with huge health issues.

The capital city Delhi areas are noticed 80% of pollution generated through transportation sector and this condition located during lockdown periods, where air quality was healthy located during lockdown by Automotive Research Association of India (ARAI) and the Energy and Resources Institute (TERI). Based on Central Pollution Control Board (CPCB) and Ministry of Environment and Forests (MoEF), motorised vehicles are generated ~ 70% of air pollutants, where industrials areas, and fossil fuel are one of the reasons for air pollution in India. The AOD was noticed high in Uttar Pradesh and neighbourhood location in 2018, where simultaneously fluctuation located in the previous four years. Nevertheless, the NO_2_ variation located high due to agricultural waste burning and related activities. The main hotspot areas are West Bengal, Uttar Pradesh, Delhi, Haryana, Punjab, Odisha, and Bihar area. Same scenarios are located in different air pollutants where AQI is also fluctuated due to COVID-19 lockdown and related restrictions. Need proper planning, management, awareness, and development for sustainable future planning and healthy life otherwise overwhelming population pressure, transportation development and industrial works are affectively influencing the air quality and increase health emergencies in future.

## Limitation and recommendation

Air quality measurement is more important factor for sustainable development of planning, human health, unplanned urban expansion and environmental degradation^8,13,62^. Many techniques are used for space-based air quality and pollutants measurement where Sentinel-5P is widely used for space-based air quality measurement and investigation^12,15,63^. But pixel data and station data can’t overlap and accurate based on the air quality index station data. The Sentinel-5P data for air pollutant measurement and MODIS for AOD application are shows that high areal pixel where station data only calculate for point data^10^, therefore large area coverage and management are not possible for air pollutants investigation. Many techniques like cloud computing-based GEE, MODIS data and station-based monitoring were used^51^. India is most populated country where megacities are mostly affected by air pollution like Delhi, Kolkata, Mumbai, Chennai, Bangalore, and industrial area. These phenomena are also triggering the air pollution in fringe location. The future research direction of this location is city-based study of air pollution, prediction of air pollution like NO_2_, CO, O_3_, and SO_2_ for development of the sustainable human life. Most of the time COVID-19 indicates the air pollution fluctuation, therefore, in this study, four decadal years’ monthly data were calculated for air pollutants analysis and development of social applicability of the investigation.

## Conclusion

Environmental phenomena and ecological disturbances are mostly facing a huge amount of disadvantages in recent times. Many anthropogenic activities like thermal variation, industrial works, transportation development, and chemical concentration are the reason for air pollutant incensement. The air pollutant like NO_2_, CO and chemical concentration like AOD is increased gradually. The earth is tired now for gradually facing anthropogenic activities, and environmental degradation. The motherland earth need rest for redevelopment of the nature and ecosystem otherwise the tired earth lead to death. The Kolkata have seven AQI motoring station where high nitrogen dioxide recorded 102 (2018), 48 (2019), 26 (2020) and 98 (2021), where Delhi AQI stations recorded 99 (2018), 49 (2019), 37 (2020), and 107 (2021). The Sulfur dioxide values of Kolkata is 29 (2018), 5 (2019), 18 (2020) and 30 (2021), where Delhi is recorded 37 (2018), 15 (2019), 14 (2020) and 38 (2021). Those results indicate the awareness and planning are much needed for protecting our environment otherwise earth surface and atmosphere are destroying every time, every second. Climate change and anthropogenic activities are the main influencing factor for air pollutant. In India, agricultural waste burning, fossil fuel burning, industrial works and vehicles are the main air pollutants development criteria where industry and transport are the main reason for urban air pollution and agricultural waste burning is the main reason for rural air pollution. Proper planning, management and development strategies can help to protect the environment otherwise climate change and air pollution will increase the health emergencies, ecological diversity and environmental degradation. This study results might be helpful for the researchers, planners, policymakers, administrators, and others stakeholders for sustainable planning for protecting the environment.

## Data Availability

Data can be provided upon request from the corresponding author.
